# Association between hospital legal constructions and medical disputes: A multi-center analysis of 130 tertiary hospitals in Hunan Province, China

**DOI:** 10.3389/fpubh.2022.993946

**Published:** 2022-09-07

**Authors:** Min Yi, Yanlin Cao, Yujin Zhou, Yuebin Cao, Xueqian Zheng, Jiangjun Wang, Wei Chen, Liangyu Wei, Ke Zhang

**Affiliations:** ^1^Institute of Medical Information, Chinese Academy of Medical Sciences and Peking Union Medical College, Beijing, China; ^2^Health Commission of Hunan Province, Changsha, China; ^3^Chinese Hospital Association Medical Legality Specialized Committee, Beijing, China; ^4^China-Japan Friendship Hospital, Beijing, China; ^5^Beijing Jishuitan Hospital, Beijing, China; ^6^Beijing Hospital, Beijing, China

**Keywords:** administrators, medical disputes, hospital legal constructions, rule of law, risk factors, nomogram, web calculator

## Abstract

**Background:**

Medical disputes are common in hospitals and a major challenge for the operations of medical institutions. However, few studies have looked into the association between medical disputes and hospital legal constructions. The purpose of the study was to investigate the relationship between hospital legal constructions and medical disputes, and it also aimed to develop a nomogram to estimate the likelihood of medical disputes.

**Methods:**

Between July and September 2021, 2,716 administrators from 130 hospitals were enrolled for analysis. The study collected seventeen variables for examination. To establish a nomogram, administrators were randomly split into a training group (*n* = 1,358) and a validation group (*n* = 1,358) with a 50:50 ratio. The nomogram was developed using data from participants in the training group, and it was validated in the validation group. The nomogram contained significant variables that were linked to medical disputes and were identified by multivariate analysis. The nomogram's predictive performance was assessed utilizing discriminative and calibrating ability. A web calculator was developed to be conducive to model utility.

**Results:**

Medical disputes were observed in 41.53% (1,128/2,716) of participants. Five characteristics, including male gender, higher professional ranks, longer length of service, worse understanding of the hospital charters, and worse construction status of hospital rule of law, were significantly associated with more medical disputes based on the multivariate analysis. As a result, these variables were included in the nomogram development. The AUROC was 0.67 [95% confident interval (CI): 0.64–0.70] in the training group and 0.68 (95% CI: 0.66–0.71) in the validation group. The corresponding calibration slopes were 1.00 and 1.05, respectively, and intercepts were 0.00 and −0.06, respectively. Three risk groups were created among the participants: Those in the high-risk group experienced medical disputes 2.83 times more frequently than those in the low-risk group (*P* < 0.001).

**Conclusion:**

Medical dispute is prevailing among hospital administrators, and it can be reduced by the effective constructions of hospital rule of law. This study proposes a novel nomogram to estimate the likelihood of medical disputes specifically among administrators in tertiary hospitals, and a web calculator can be available at https://ymgarden.shinyapps.io/Predictionofmedicaldisputes/.

## Introduction

Medical dispute is a prominent global problem and has become a significant public health challenge (1, 2), particularly in China (3). Since 2016, the number of medical disputes in China has increased by almost 100,000 cases annually (4). A national study in China conducted in 2021 revealed that 31.06% of physicians had encountered medical disputes (5). Physician–patient relationship was rated as stressful by 55.73% of medical staff in the same year, according to a meta-analysis (6), which has brought significant problems to the hospital's medical order and hospital administration.

To prevent medical disputes in such circumstances, it is crucial to identify the causes of medical disputes. Medical disputes typically involve doctors, hospitals, patients, and their families (7). The majority of studies on medical disputes placed emphasis on the relationships between doctors and patients and focused on addressing the problems of medical workers (7). However, few research addressed concerns about medical disputes among hospital administrators (8). Hospital administrators are crucial in managing medical disputes because they serve as the mediators of medical disputes.

Currently, some studies suggest that medical disputes are related to medical quality, patient's misperceptions about treatments, communication skills of medical workers, and medical worker' attitudes (9–11). These factors may help to direct hospital administration in preventing medical disputes. These studies, however, were designed for healthcare professionals, such as doctors and nurses, but not specifically for hospital administrators. Furthermore, these characteristics have little to do with hospital's legal constructions. Thus, in light of hospital legal constructions, it is crucial to identify new indicators for forecasting medical, especially among administrators. In addition, developing a prediction model to estimate the risk probability of medical disputes would significantly aid early preventive measures. The prediction model, it should be noted, is a favorable mean of early detection of clinical problems and has shown promise in enhancing predictive power in a range of situations, such as emergency department triage and readmission (12–14). This strategy's benefit is that it can enhance clinical decision-making (15) and hospital management skills (16, 17). Nonetheless, to the author's knowledge, studies on prediction model of medical disputes were scarce. The transformation from passive response to active prevention of medical disputes was made possible by accurately predicting the risk likelihood of medical disputes.

Therefore, our goal was to determine the relationship between hospital legal constructions and medical disputes and to further develop a model to estimate the likelihood of medical disputes specifically among hospital administrators. Due to its individualized forecasts and user-friendly interface, nomogram, which combines multiple variables into a straightforward graphical representation, is frequently utilized in a range of sectors, such as the prediction of patient satisfaction for hospital management (18–20). Thus, to accurately and individually assess the possibility of encountering medical disputes, particularly among administrators, the prediction model was presented as the format of nomogram. In addition, a web calculator was also developed to promote model utility.

## Methods

### Participants and study design

This study conducted a cross-sectional survey from July to September 2021. We constructed a survey (Supplementary File 1) following an extensive assessment of the literature and successive discussions with twelve experts, including public health specialists, experts in healthcare service administration, and experts in administrative departments. The survey mainly collected participant's demographics, occupations, hospital-related data, the construction status of hospital rule of law, and administrator's knowledge on medical laws. To conduct the study, the Health Commission of Hunan Province (a provincial health administrative department) used WeChat software (an instant messaging software) to distribute an online survey to lower-level health administrative departments. The survey was then forwarded to 130 tertiary hospitals in the Hunan province of China. All of the administrators were requested to voluntarily complete the survey, and it could only be submitted if all of the questions were answered according to their actual situations. As a result, the study could ensure the accuracy and integrity of the data.

**Figure 1:**
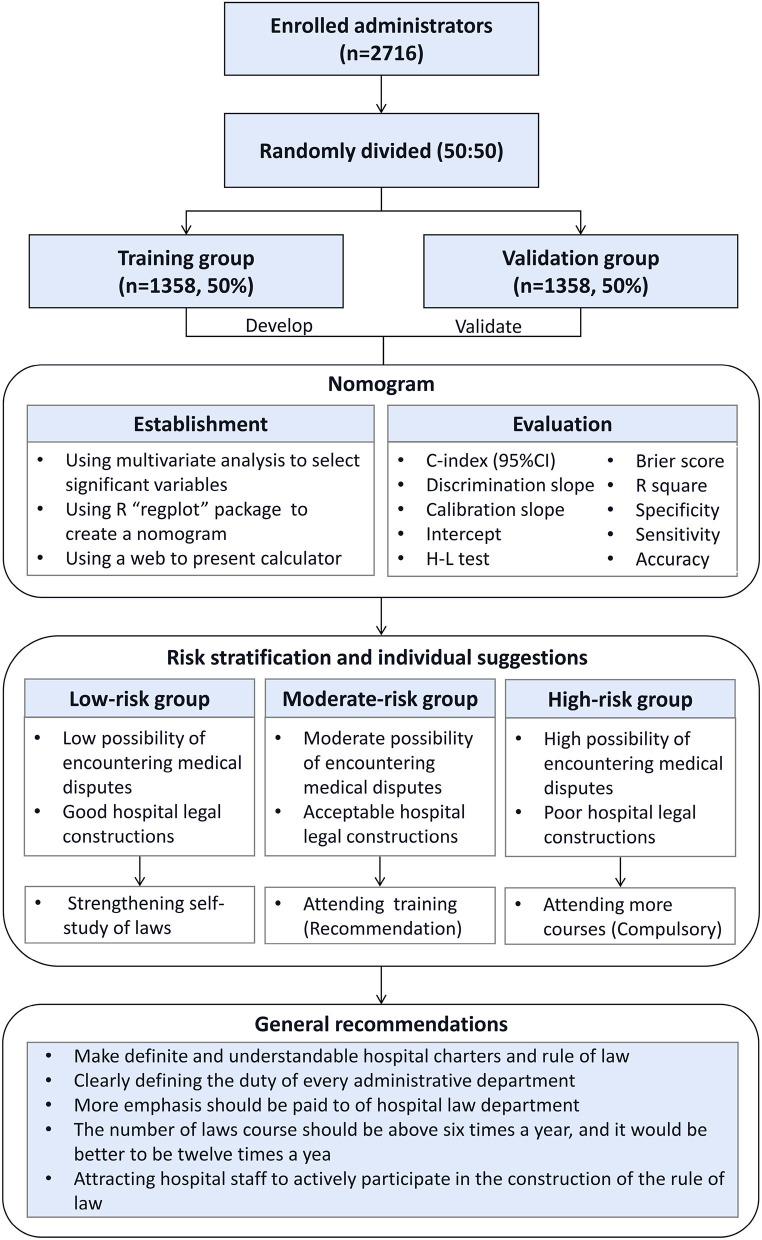
Patient's flowchart and study design.

In this study, hospital administrators primarily refer to hospital administration employees, such as members of the medical, nursing, dispute resolution, and outpatient departments. If a participant was (1) not administrative staff, such as healthcare workers, medical students, cleaning staff, clerks, and securities, (2) not employed by a tertiary hospital, (3) objected to taking in the survey, and (4) unable to cooperate for any other reasons, they were excluded from the study. In the end, a total of 2,716 valid questionnaires were obtained for analysis based on the above inclusive and exclusive criteria. The entire cohort of participants was randomly split into two groups based on a 50:50 ratio. Participants in the training group (*n* = 1,358) were used to establish the nomogram, and participants in the validation group (*n* = 1,358) underwent internal validation of the nomogram. The flowchart and design of this study are summarized in Figure 1.

The study protocol was approved by the Hunan Provincial Health Commission, a government official health management agency in China. All 130 tertiary hospitals had understood the whole content of the survey and supported this project. The survey was voluntary, anonymous, and did not ask for any personal information from the participants. All participants were requested to provide their informed consent prior to participating in the survey. The study procedure adheres to the Helsinki Declaration of 2013.

### Variables and their definitions

The study collected seventeen potential characteristics for the purpose of evaluating their ability to predict medical disputes among hospital administrators. These characteristics included participant's demographics (gender, age, length of service, and working in a department directly providing window services to patients), occupations (professional ranks), hospital-related data (hospital type, hospital category, tertiary hospital level, and hospital located in provincial capital), the construction status of hospital rule of law (establishment of law frameworks in the hospital, number of laws or regulations training organized by hospitals each year, number of participating in the training of laws or regulations each year, establishment of a performance appraisal system related to the rule of law, and the construction status of hospital rule of law), and administrator's knowledge on medical laws (understanding of the duty of hospital law department, understanding of hospital charters, and understanding of the contents of legitimacy review in the hospital).

All of these features were self-reported based on participant's actual situation. Hospital charters are law frameworks for hospitals made up of state legislation, administrative norms, and hospital rules and regulations. Performance appraisal system mainly refers to performance of job responsibilities, completion of annual work, and work attendance, which can directly affect earnings, awards, and academic or job title assessments. The contents of legitimacy review refer to major policy measures for hospital, all kinds of master plans, major hospital investment projects and the disposal of state-owned assets, or other major matters that require decision-making by the hospital. Medical disputes include doctor–patient disputes, disputes between patients or their families and hospital staff, and other disputes related to medical works.

### Construction of the nomogram

The multivariate analysis was performed to evaluate the ability of the above seventeen potential risk factors for predicting medical disputes among administrators in the training group. Significant variables were included for developing the nomogram. The nomogram was developed using the “regplot” package of R programing language. To further increase model usability, a web calculator was developed to display the nomogram. The web calculator included summaries of model information, model predictors, and estimated probability of medical disputes.

### Evaluation of the nomogram

The predictive effectiveness of the nomogram was evaluated both in the training and validation groups. The predictive metrics mainly included discrimination and calibration (21). Discrimination of the model referred to the capability to distinguish participants with and without medical disputes, and its metrics mainly included area under the receiver operating characteristic (AUROC) curve and discrimination slope. The mean difference in predicted probabilities between hospital administrators who have and did not have medical disputes is known as the discrimination slope.

The consistency between the predicted probability of medical disputes and actual observed probability of medical disputes, which was assessed based on calibration curves and goodness-of-fit statistics, was characterized as the calibrating ability of the nomogram. Based on calibration curves, calibration slope and intercept were calculated. The ideal calibration slope and intercept values show perfect consistency when the two are close to 1.00 and near to 0.00, respectively. Goodness-of-fit statistics were evaluated using the Hosmer and Lemeshow test, and a *P* > 0.05 indicates favorable calibration.

In addition, Brier score, Brier_scaled_ score, and *R*^2^ were also evaluated in the study. Brier score was defined as the mean square between predicted probability and the outcome (0 indicating participants without medical disputes and 1 representing participants with medical disputes). A decision curve analysis was also conducted to assess the nomogram's clinical usefulness. In the study, specificity, sensitivity, and accuracy of the model were also calculated and presented.

### Classification of risk using the nomogram

This study used the optimal threshold to classify participants into three risk groups. Participants with predicted probability less than the optimal threshold were assigned to the low-risk group, and participants with predicted probability greater than twice the optimal threshold were assigned to the high-risk group. The remaining participants were categorized as belonging to a moderate-risk group. The predicted and actual probabilities of medical disputes in each risk category were computed and compared.

### Statistical analysis

In the study, variables were expressed as proportions to better build nomogram. According to whether participants had medical disputes, a subgroup analysis of participants was conducted, and a comparison of characteristics was made using the chi-square test, supplemented by continuous adjusted chi-square test or Fisher's exact test. Univariate and multivariate analyses were used to identify potential risk variables for predicting medical disputes. Statistical analysis was performed using SPSS (version 21) and R programming language (version 4.1.2), and all data visualizations were carried out using R programming language (version 4.1.2). *P* < 0.05 (two-tailed) were defined as statistically significant.

## Results

### Basic characteristics, hospital levels, and the construction status of hospital rule of law

A total of 2,716 hospital administrators were enrolled for analysis in the study, and 64.62% participants were women. The professional rank of administrators was mainly concentrated on the intermediate (37.3%). Of all included participants, 38.11% had more than 21 years of service. Regarding the construction status of hospital rule of law, the results seemed favorable, because 97.97% of the administrators' hospitals have established legal framework, and 98.23% of the participants' hospitals have established a performance appraisal system related to the rule of law.

However, although the majority of participants claimed to understand the duty of hospital law department, hospital charters, and the contents of legitimacy review in the hospital, only about half of participants had a completely clear understanding of these contents, indicating that their understanding of hospital rule of law still needed to be improved. In addition, up to 30.67% of the participants' hospitals conducted training courses on laws or regulations for no more than once a year, and 50.15% of participants attended training courses on laws or regulations just once or twice a year on average. The findings indicated that the participant's inadequate comprehension of hospital rule of law may have been caused by the training sessions' short duration. Therefore, only 53.39% thought that the rule of law being established in their hospitals was in a very satisfactory way, and among all participants, up to 41.53% (1,128/2,716) of participants reported encountering medical disputes at work. Table 1 provides a summary of additional information on the sociodemographic and hospital rule of law construction characteristics of participants.

**Table 1:** Administrator's basic characteristics, hospital levels, and the construction status of the rule of law in their hospitals.

**Characteristics**	**Participants (*n* = 2,716)**
**Gender**	
Male	35.38% (961/2,716)
Female	64.62% (1,755/2,716)
**Age (years)**	
<30	15.87% (431/2,716)
30–39	36.45% (990/2,716)
40–49	34.32% (932/2,716)
≥50	13.37% (363/2,716)
**Professional ranks**	
Senior	3.39% (92/2,716)
Deputy senior	20.29% (551/2,716)
Intermediate	37.3% (1,013/2,716)
Junior	24.82% (674/2,716)
None	14.21% (386/2,716)
**Length of service (years)**	
<6	14.25% (387/2,716)
6–10	17.86% (485/2,716)
11–15	17.19% (467/2,716)
16–20	12.59% (342/2,716)
>20	38.11% (1,035/2,716)
**Hospital type**	
Public	95.58% (2,596/2,716)
Private	4.42% (120/2,716)
**Hospital category**	
General	51.44% (1,397/2,716)
TCM general	29.12% (791/2,716)
Specialized	18.93% (514/2,716)
Other	0.51% (14/2,716)
**Tertiary hospital level**	
Class A	56.19% (1,526/2,716)
Class B	8.95% (243/2,716)
Other	34.87% (947/2,716)
**Hospital located in provincial capital**	
Yes	14.91% (405/2,716)
No	85.09% (2,311/2,716)
**Working in a department directly providing window services to patients**	
Yes	37.41% (1,016/2,716)
No	62.59% (1,700/2,716)
**Establishment of law frameworks in the hospital**	
Yes	97.97% (2,661/2,716)
No	0.29% (8/2,716)
Not clear	1.73% (47/2,716)
**Understanding of the duty of hospital law department**	
Perfectly clear	50.55% (1,373/2,716)
Clear	45.07% (1,224/2,716)
Slightly clear	3.76% (102/2,716)
Not at all	0.63% (17/2,716)
**Number of laws or regulations training organized by participant's hospital each year**	
≤ 1	30.67% (833/2,716)
2–3	25% (679/2,716)
4–6	14.06% (382/2,716)
7–10	16.72% (454/2,716)
≥11	13.55% (368/2,716)
**Number of participating in the training of laws or regulations each year**	
0	3.28% (89/2,716)
1–2	50.15% (1,362/2,716)
3–4	27.72% (753/27,16)
≥5	18.85% (512/2,716)
**Understanding of hospital charters**	
Perfectly clear	52.17% (1,417/2,716)
Clear	45.73% (1,242/2,716)
Slightly clear	1.66% (45/2,716)
Not at all	0.18% (5/2,716)
No hospital charters	0.26% (7/2,716)
**Establishment of a performance appraisal system related to the rule of law in the hospital**	
Yes	98.23% (2,668/2,716)
No	1.77% (48/2,716)
**Understanding of the contents of legitimacy review in the hospital**	
Perfectly clear	48.67% (1,322/2,716)
Clear	45.84% (1,245/2,716)
Slightly clear	5.04% (137/2,716)
Not at all	0.44% (12/2,716)
**Construction status of hospital rule of law**	
Very good	53.39% (1,450/2,716)
Good	36.93% (1,003/2,716)
Neither good nor bad	9.46% (257/2,716)
Bad	0.15% (4/2,716)
Very bad	0.07% (2/2,716)
**Encountered medical disputes at work**	
No	58.47% (1,588/2,716)
Yes	41.53% (1,128/2,716)

TCM, Traditional Chinese medicine.

### Participants with and without medical disputes: A comparison

Participants who encountered more medical disputes tended to be male (*P* < 0.001), older age (*P* < 0.001), higher professional rank (*P* < 0.001), longer length of service (*P* < 0.001), general or specialized hospitals (*P* = 0.018), worse understanding of the duty of hospital law department (*P* = 0.002), lower number of laws or regulations training organized by hospitals each year (*P* < 0.001), lower number of participating in the training of laws or regulations each year (*P* < 0.001), worse understanding of hospital charters (*P* < 0.001), worse understanding of the contents of legitimacy review in the hospital (*P* < 0.001), and the worse construction status of hospital rule of law (*P* < 0.001), as compared to participants without medical disputes. Table 2 provides a summary of more details.

**Table 2:** Comparison of characteristics between administrators with and without medical disputes.

**Characteristics**	**Incidence (*n* = 2,716)**	**Medical disputes**		***P***
		**Yes (*n* = 1,128)**	**No (*n* = 1,588)**	
**Gender**				
Male	49.84% (479/961)	42.46% (479/1,128)	30.35% (482/1,588)	<0.001
Female	36.98% (649/1,755)	57.54% (649/1,128)	69.65% (1,106/1,588)	
**Age (years)**				
<30	33.18% (143/431)	12.68% (143/1,128)	18.14% (288/1,588)	<0.001
30–39	37.68% (373/990)	33.07% (373/1,128)	38.85% (617/1,588)	
40–49	46.46% (433/932)	38.39% (433/1,128)	31.42% (499/1,588)	
≥50	49.31% (179/363)	15.87% (179/1,128)	11.59% (184/1,588)	
**Professional ranks**				
Senior	59.78% (55/92)	4.88% (55/1,128)	2.33% (37/1,588)	<0.001
Deputy senior	52.27% (288/551)	25.53% (288/1,128)	16.56% (263/1,588)	
Intermediate	42.65% (432/1,013)	38.30% (432/1,128)	36.59% (581/1,588)	
Junior	32.79% (221/674)	19.59% (221/1,128)	28.53% (453/1,588)	
None	34.20% (132/386)	11.70% (132/1,128)	15.99% (254/1,588)	
**Length of service (years)**				
<6	31.27% (121/387)	10.73% (121/1,128)	16.75% (266/1,588)	<0.001
6–10	37.32% (181/485)	16.05% (181/1,128)	19.14% (304/1,588)	
11–15	36.40% (170/467)	15.07% (170/1,128)	18.70% (297/1,588)	
16–20	45.03% (154/342)	13.65% (154/1,128)	11.84% (188/1,588)	
>20	48.50% (502/1,035)	44.50% (502/1,128)	33.56% (533/1,588)	
**Hospital type**				
Public	41.29% (1,072/2,596)	95.04% (1,072/1,128)	95.97% (1,524/1,588)	0.243
Private	46.67% (56/120)	4.96% (56/1,128)	4.03% (64/1,588)	
**Hospital category**				
General	43.31% (605/1,397)	53.63% (605/1,128)	49.87% (792/1,588)	0.018
TCM general	37.17% (294/791)	26.06% (294/1,128)	31.30% (497/1,588)	
Specialized	43.77% (225/514)	19.95% (225/1,128)	18.20% (289/1,588)	
Other	28.57% (4/14)	0.35% (4/1,128)	0.63% (10/1,588)	
**Tertiary hospital level**				
Class A	40.76% (622/1,526)	55.14% (622/1,128)	56.93% (904/1,588)	0.167
Class B	37.86% (92/243)	8.16% (92/1,128)	9.51% (151/1,588)	
Other	43.72% (414/947)	36.70% (414/1,128)	33.56% (533/1,588)	
**Hospital located in provincial capital**				
Yes	45.19% (183/405)	16.22% (183/1,128)	13.98% (222/1,588)	0.106
No	40.89% (945/2,311)	83.78% (945/1,128)	86.02% (1,366/1,588)	
**Working in a department directly providing window services to patients**				
Yes	42.62% (433/1,016)	38.39% (433/1,128)	36.71% (583/1,588)	0.374
No	40.88% (695/1,700)	61.61% (695/1,128)	63.29% (1,005/1,588)	
**Establishment of law in the hospital**				
Yes	41.53% (1,105/2,661)	97.96% (1,105/1,128)	97.98% (1,556/1,588)	0.968
No	37.50% (3/8)	0.27% (3/1,128)	0.31% (5/1,588)	
Not clear	42.55% (20/47)	1.77% (20/1,128)	1.70% (27/1,588)	
**Understanding of the duty of hospital law department**				
Perfectly clear	38.09% (523/1,373)	46.37% (523/1,128)	53.53% (850/1,588)	0.002
Clear	44.77% (548/1,224)	48.58% (548/1,128)	42.57% (676/1,588)	
Slightly clear	46.08% (47/102)	4.17% (47/1,128)	3.46% (55/1,588)	
Not at all	58.82% (10/17)	0.89% (10/1,128)	0.44% (7/1,588)	
**Number of laws or regulations training organized by participant's hospitals each year**				
≤ 1	45.14% (376/833)	33.33% (376/1,128)	28.78% (457/1,588)	<0.001
2–3	46.10% (313/679)	27.75% (313/1,128)	23.05% (366/1,588)	
4–6	46.07% (176/382)	15.60% (176/1,128)	12.97% (206/1,588)	
7–10	38.11% (173/454)	15.34% (173/1,128)	17.70% (281/1,588)	
≥11	24.46% (90/368)	7.98% (90/1,128)	17.51% (278/1,588)	
**Number of participating in the training of laws or regulations each year**				
0	40.45% (36/89)	3.19% (36/1,128)	3.34% (53/1,588)	<0.001
1–2	44.35% (604/1,362)	53.55% (604/1,128)	47.73% (758/1,588)	
3–4	43.29% (326/753)	28.90% (326/1,128)	26.89% (427/1,588)	
≥5	31.64% (162/512)	14.36% (162/1,128)	22.04% (350/1,588)	
**Understanding of hospital charters**				
Perfectly clear	35.71% (506/1,417)	44.86% (506/1,128)	57.37% (911/1,588)	<0.001
Clear	47.58% (591/1,242)	52.39% (591/1,128)	40.99% (651/1,588)	
Slightly clear	48.89% (22/45)	1.95% (22/1,128)	1.45% (23/1,588)	
Not at all	60.00% (3/5)	0.27% (3/1,128)	0.13% (2/1,588)	
No hospital charters	85.71% (6/7)	0.53% (6/1,128)	0.06% (1/1,588)	
**Establishment of a performance appraisal system related to the rule of law in the hospital**				
Yes	41.34% (1,103/2,668)	97.78% (1,103/1,128)	98.55% (1,565/1,588)	0.134
No	52.08% (25/48)	2.22% (25/1,128)	1.45% (23/1,588)	
**Understanding of the contents of legitimacy review in the hospital**				
Perfectly clear	36.54% (483/1,322)	42.82% (483/1,128)	52.83% (839/1,588)	<0.001
Clear	47.55% (592/1,245)	52.48% (592/1,128)	41.12% (653/1,588)	
Slightly clear	33.58% (46/137)	4.08% (46/1,128)	5.73% (91/1,588)	
Not at all	58.33% (7/12)	0.62% (7/1,128)	0.31% (5/1,588)	
**Construction status of hospital rule of law**				
Very good	31.93% (463/1,450)	41.05% (463/1,128)	62.15% (987/1,588)	<0.001
Good	51.05% (512/1,003)	45.39% (512/1,128)	30.92% (491/1,588)	
Neither good nor bad	57.59% (148/257)	13.12% (148/1,128)	6.86% (109/1,588)	
Bad	75.00% (3/4)	0.27% (3/1,128)	0.06% (1/1,588)	
Very bad	100.00% (2/2)	0.18% (2/1,128)	0.00% (0/1,588)	

TCM, Traditional Chinese medicine.

**Figure 2:**
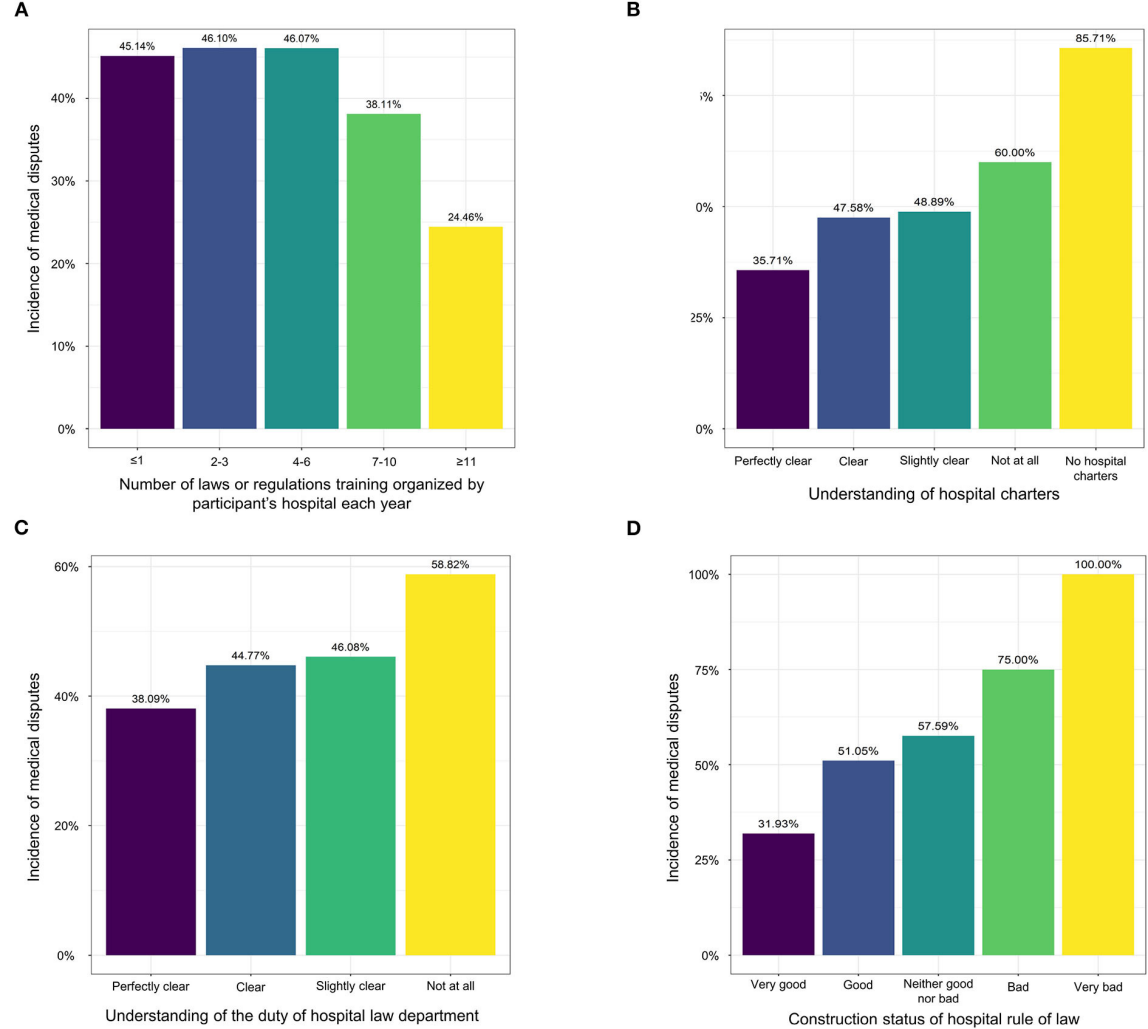
Histograms of the incidence of medical disputes in relation to hospital legal constructions and administrator's knowledge on hospital rule of law. **(A)** Number of laws or regulations training organized by participant's hospitals each year; **(B)** understanding of hospital charters; **(C)** understanding of duty of hospital law department; **(D)** construction status of hospital rule of law.

To further explain, based on the findings, the study also showed that the incidence of medical disputes remained stable (45.14–46.10%) when the number of laws or regulations training courses that organized by hospitals ranged from zero to six times each year, but it could decrease to 38.11% when the number was above six and <11 times a year, and it could further decline to only 24.46% when hospitals set up laws or regulations training course for eleven or above times a year (Figure 2A). A similar pattern was also seen in terms of the number of attending the training of laws or regulations a year in participants.

Regarding participant's knowledge on hospital rule of law, only when they had a perfect clear understanding of hospital charters (Figure 2B), the duty of hospital law department (Figure 2C), the contents of legitimacy review, the incidence of medical disputes could remarkably decline. So was the construction status of hospital rule of law. In detail, the incidence of medical disputes was only 31.93% among hospitals with very good construction of hospital rule of law, but it increased to over 50.00% and even 100.00% among hospitals with other construction status of hospital rule or law (Figure 2D).

### Development of the nomogram

Participants were randomly divided into the training and validation groups with a ratio of 50:50. The distribution of all characteristics was comparable between the two groups. In the training group, according to the multiple logistic regression analysis, five variables were significantly associated with medical disputes and were included in the nomogram, including gender, professional ranks, length of service, understanding of hospital charters, and the construction status of hospital rule of law (Table 3).

**Table 3:** Multivariate logistic regression analyses of potential risk variables for hospital administrators encountered medical disputes at work in the training group.

**Characteristics**	**Incidence (*n* = 1,358)**	**Univariate logistic regression**		**Multivariate logistic regression**	
		**OR (95% CI)**	***P***	**OR (95% CI)**	***P***
**Gender**					
Male	51.46% (246/478)	0.571 (0.456–0.716)	<0.001	0.538 (0.424–0.684)	<0.001
Female	37.73% (332/880)				
**Age (years)**					
<30	34.62% (72/208)	1.269 (1.127–1.429)	<0.001	0.844 (0.673–1.059)	0.143
30–39	38.14% (185/485)				
40–49	48.20% (228/473)				
≥50	48.44% (93/192)				
**Professional ranks**					
Senior	56.14% (32/57)	0.756 (0.680–0.841)	<0.001	0.776 (0.675–0.891)	<0.001
Deputy senior	52.28% (149/285)				
Intermediate	43.44% (222/511)				
Junior	35.19% (120/341)				
None	33.54% (55/164)				
**Length of service (years)**					
<6	32.40% (58/179)	1.200 (1.114–1.292)	<0.001	1.217 (1.047–1.416)	0.011
6–10	38.33% (92/240)				
11–15	35.81% (82/229)				
16–20	44.97% (76/169)				
>20	49.91% (270/541)				
**Hospital type**					
Public	42.06% (543/1,291)	1.507 (0.921–2.464)	0.102	1.647 (0.947–2.864)	0.077
Private	52.24% (35/67)				
**Hospital category**					
General	44.96% (317/705)	0.935 (0.816–1.072)	0.337	0.927 (0.798–1.077)	0.323
TCM general	37.18% (145/390)				
Specialized	44.53% (114/256)				
Other	28.57% (2/7)				
**Tertiary hospital level**					
Class A	41.58% (316/760)	1.075 (0.958–1.206)	0.221	1.010 (0.888–1.149)	0.875
Class B	37.90% (47/124)				
Other	45.36% (215/474)				
**Hospital located in provincial capital**					
Yes	45.15% (93/206)	0.884 (0.656–1.191)	0.416	0.881 (0.638–1.217)	0.442
No	42.10% (485/1,152)				
**Working in a department directly providing window services to patients**					
Yes	45.31% (232/512)	0.835 (0.669–1.042)	0.111	0.839 (0.664–1.061)	0.142
No	40.90% (346/846)				
**Establishment of law in the hospital**					
Yes	42.49% (566/1,332)	1.075 (0.720–1.606)	0.723	1.071 (0.675–1.697)	0.772
No	50.00% (1/2)				
Not clear	45.83% (11/24)				
**Understanding of the duty of hospital law department**					
Perfectly clear	38.91% (272/699)	1.276 (1.065–1.530)	0.008	0.829 (0.632–1.086)	0.173
Clear	46.36% (280/604)				
Slightly clear	46.81% (22/47)				
Not at all	50.00% (4/8)				
**Number of laws or regulations training organized by participant's hospital each year**					
≤ 1	46.70% (198/424)	0.864 (0.800–0.933)	<0.001	0.975 (0.875–1.086)	0.640
2–3	41.76% (142/340)				
4–6	52.54% (93/177)				
7–10	42.37% (100/236)				
≥11	24.86% (45/181)				
**Number of participating in the training of laws or regulations each year**					
0	51.11% (23/45)	0.812 (0.711–0.927)	0.002	0.965 (0.793–1.174)	0.721
1–2	44.81% (298/665)				
3–4	43.77% (172/393)				
≥5	33.33% (85/255)				
**Understanding of hospital charters**					
Perfectly clear	36.38% (259/712)	1.600 (1.320–1.940)	<0.001	1.377 (1.043–1.818)	0.024
Clear	49.02% (301/614)				
Slightly clear	53.85% (14/26)				
Not at all	66.67% (2/3)				
No hospital charters	66.67% (2/3)				
**Establishment of a performance appraisal system related to the rule of law in the hospital**					
Yes	42.37% (569/1,343)	2.040 (0.722–5.765)	0.178	1.158 (0.383–3.499)	0.795
No	60.00% (9/15)				
**Understanding of the contents of legitimacy review in the hospital**					
Perfectly clear	37.29% (248/665)	1.341(1.122–1.602)	0.001	0.883 (0.674–1.157)	0.368
Clear	48.55% (301/620)				
Slightly clear	35.29% (24/68)				
Not at all	100.00% (5/5)				
**Construction status of hospital rule of law**					
Very good	34.01% (250/735)	1.788 (1.515–2.110)	<0.001	1.793 (1.418–2.267)	<0.001
Good	51.11% (253/495)				
Neither good nor bad	57.94% (73/126)				
Bad	0.00% (0/0)				
Very bad	100.00% (2/2)				

TCM, Traditional Chinese medicine; OR, odds ratio; CI, confident interval.

**Figure 3:**
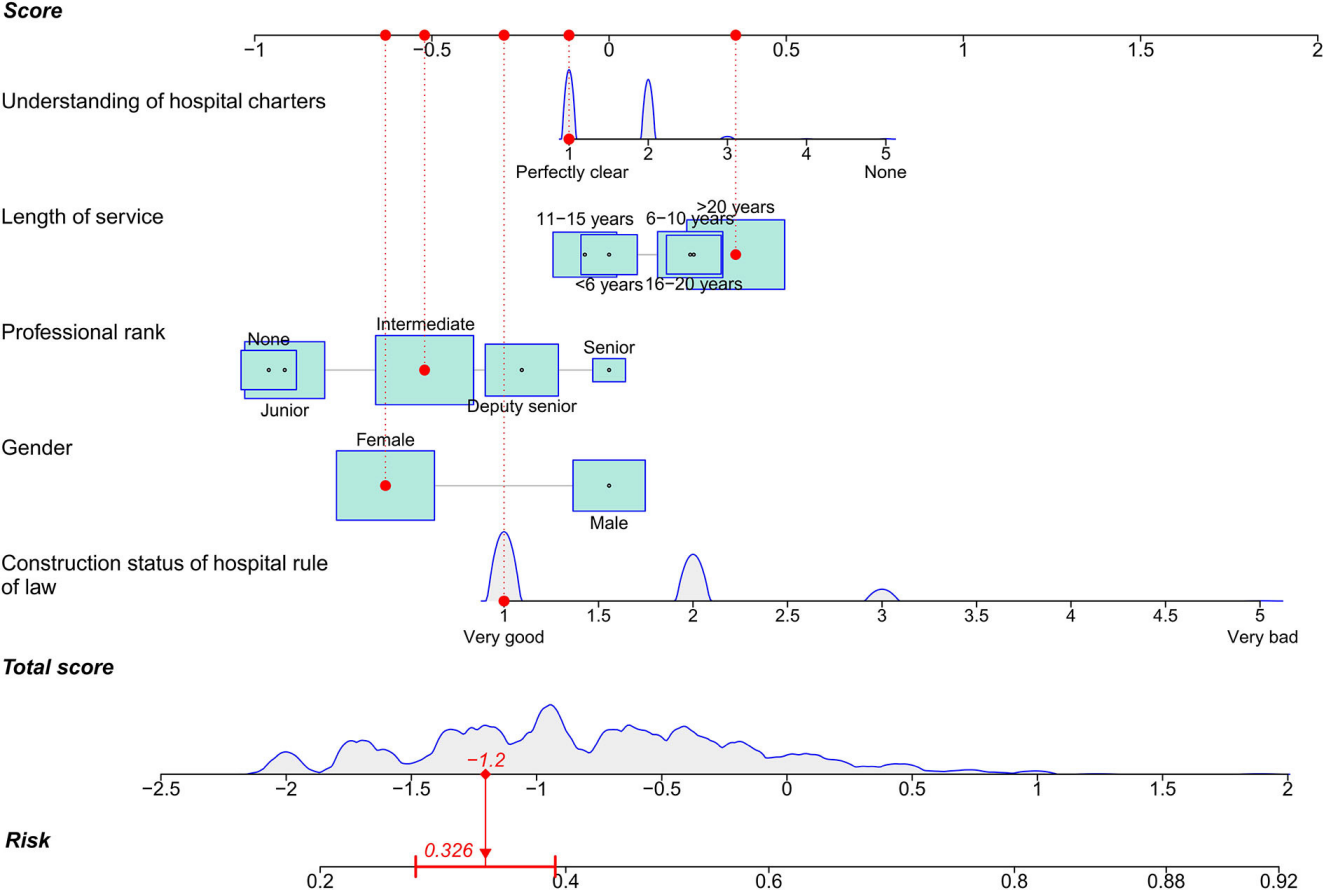
Nomogram to predict the likelihood of medical disputes. An illustration of using the nomogram is provided: Each feature can obtain a score by referring to score axis, and the total score is the sum of all the features. The final risk probability of medical disputes can be determined by referring to risk axis from overall score axis. Continuous features are presented as density curve, and proportion features are depicted as boxes, with larger boxes denoting higher proportions.

Furthermore, the study developed a nomogram to estimate the risk probability of medical disputes among tertiary hospital administrators based on the significant features (Figure 3). As depicted in the nomogram, predictors like gender were portrayed as boxes, with the size of the box indicating proportion. Predictors such as the construction status of hospital rule of law were summarized as density plot. An illustration of using the nomogram to estimate the likelihood of experiencing medical disputes among administrators was provided. In the described example, a female administrator with a middle rank had a perfectly clear understanding of hospital charters, had been employed for more than 20 years, and was in a hospital where the rule of law was well-established. Each predictor's score could be determined by referring to the score axis, and overall score was the sum of each predictor's score (-1.2). At last, the predicted probability of medical disputes (32.6%) could be obtained by downing to risk axis from the total score axis.

In addition, the study established a Shiny app-based web calculator to help the model be more convenient for users to use, and the website can be found at https://ymgarden.shinyapps.io/Predictionofmedicaldisputes/. It consists of a variable selection part and a result presenting component on the website. Users are able to choose each feature in accordance with their current situation before clicking “Predict” bottom. The website can also automatically display graphical, numerical, and model summaries for the specified scenario. A thorough predicted probability of medical disputes for the specific participant is provided in the numerical summary.

### Validation of the nomogram

**Figure 4:**
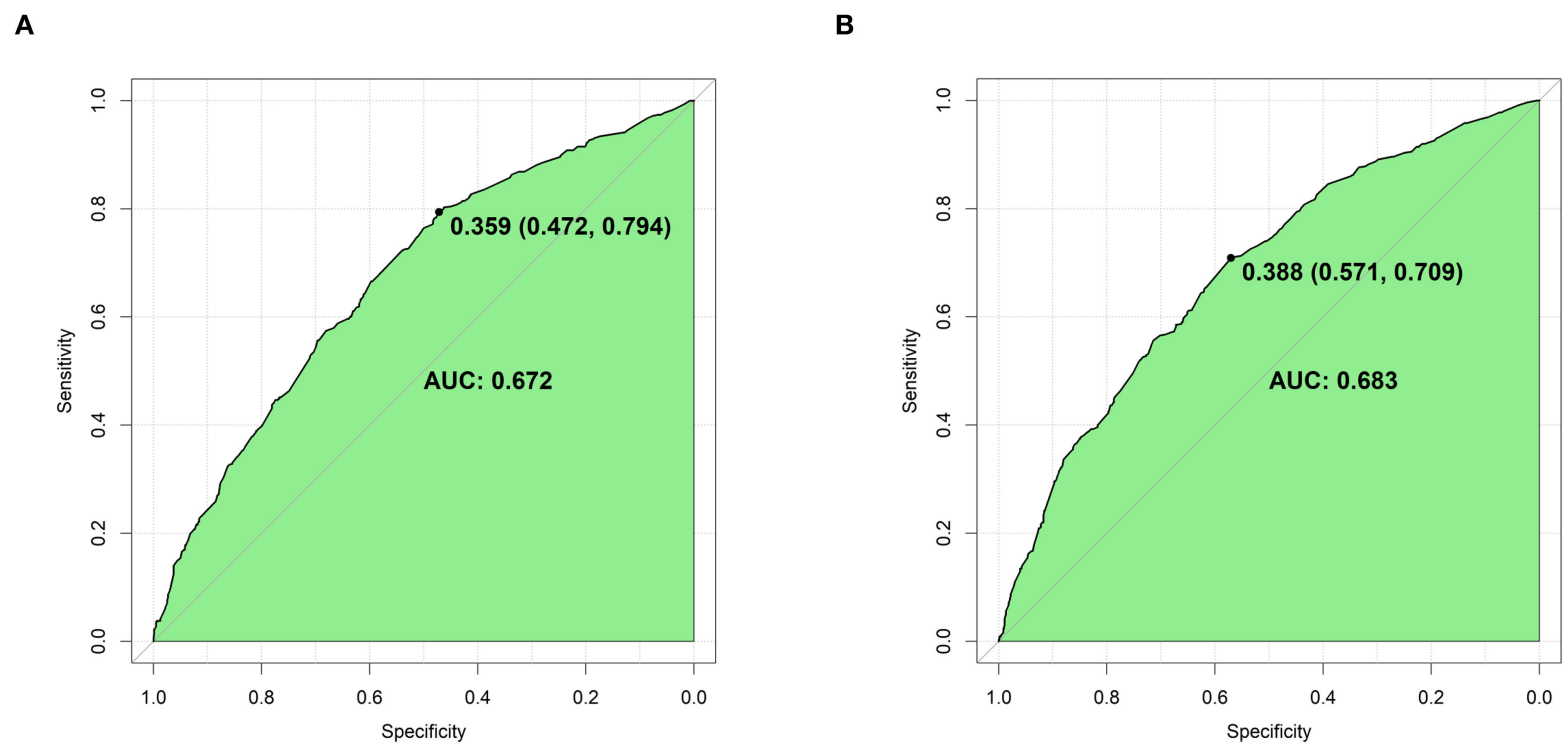
Area under receiver operation curve (AUROC) for the nomogram in the two groups. **(A)** The training group (AUROC = 0.67); **(B)** the validation group (AURCO = 0.68). The curve was plotted with one specificity against sensitivity.

**Figure 5:**
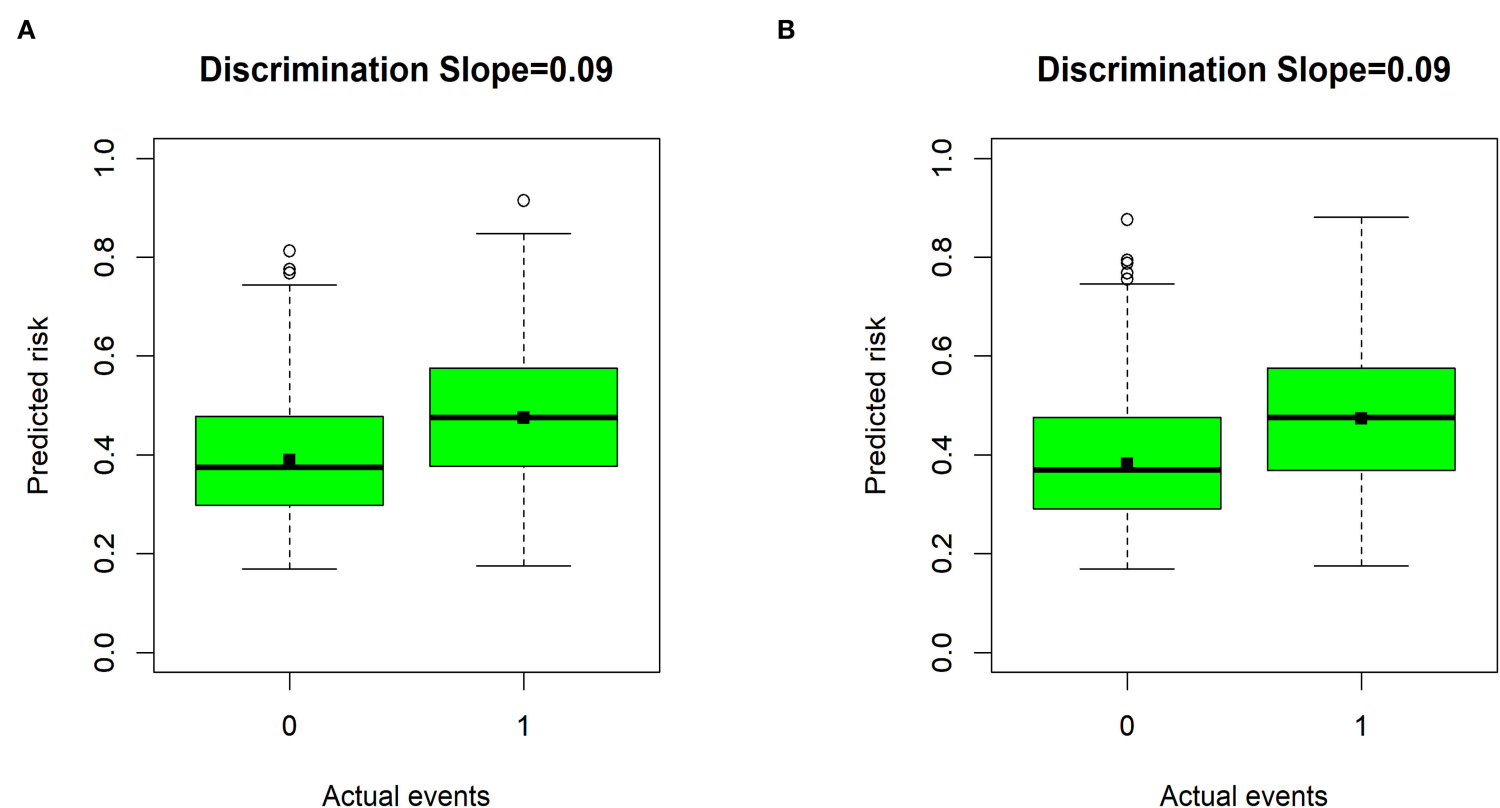
Discrimination slopes for the nomogram in the two groups. **(A)** The training group; **(B)** the validation group. “0” indicates participants without medical disputes, and “1” indicates participants with medical disputes.

**Figure 6:**
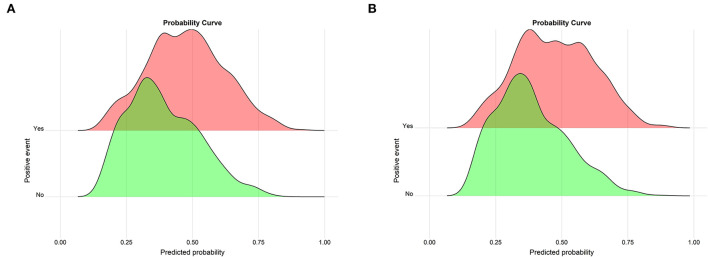
Probability curve for the nomogram in the two groups. **(A)** The training group; **(B)** the validation group. Probability curve is drawn by predicted probability against events. The green curve indicates negative events (participants without medical disputes), whereas the red curve indicates positive events (participants with medical disputes). A greater distance between the peaks of the red and green curves on the nomogram implies better separation.

**Figure 7:**
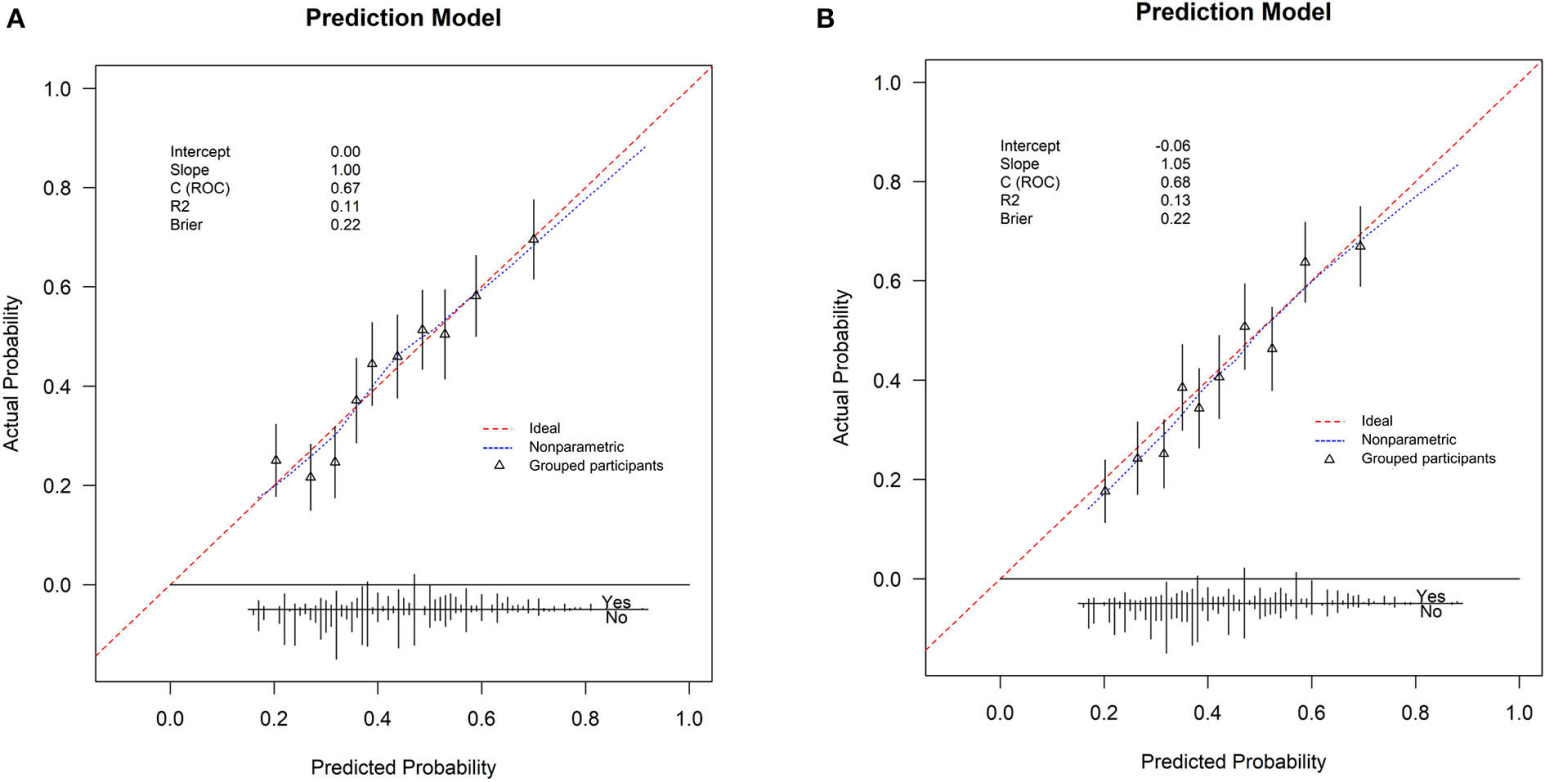
Calibration curve for the nomogram in the two groups. **(A)** The training group; **(B)** the validation group. Calibration curve is plotted by predicted probability with observed probability. The red line indicates perfect consistency between predicted and observed probability, and the blue line indicates the calibrating line for the nomogram. The closer the blue line is to the red line, the better the calibration ability of the nomogram.

**Figure 8:**
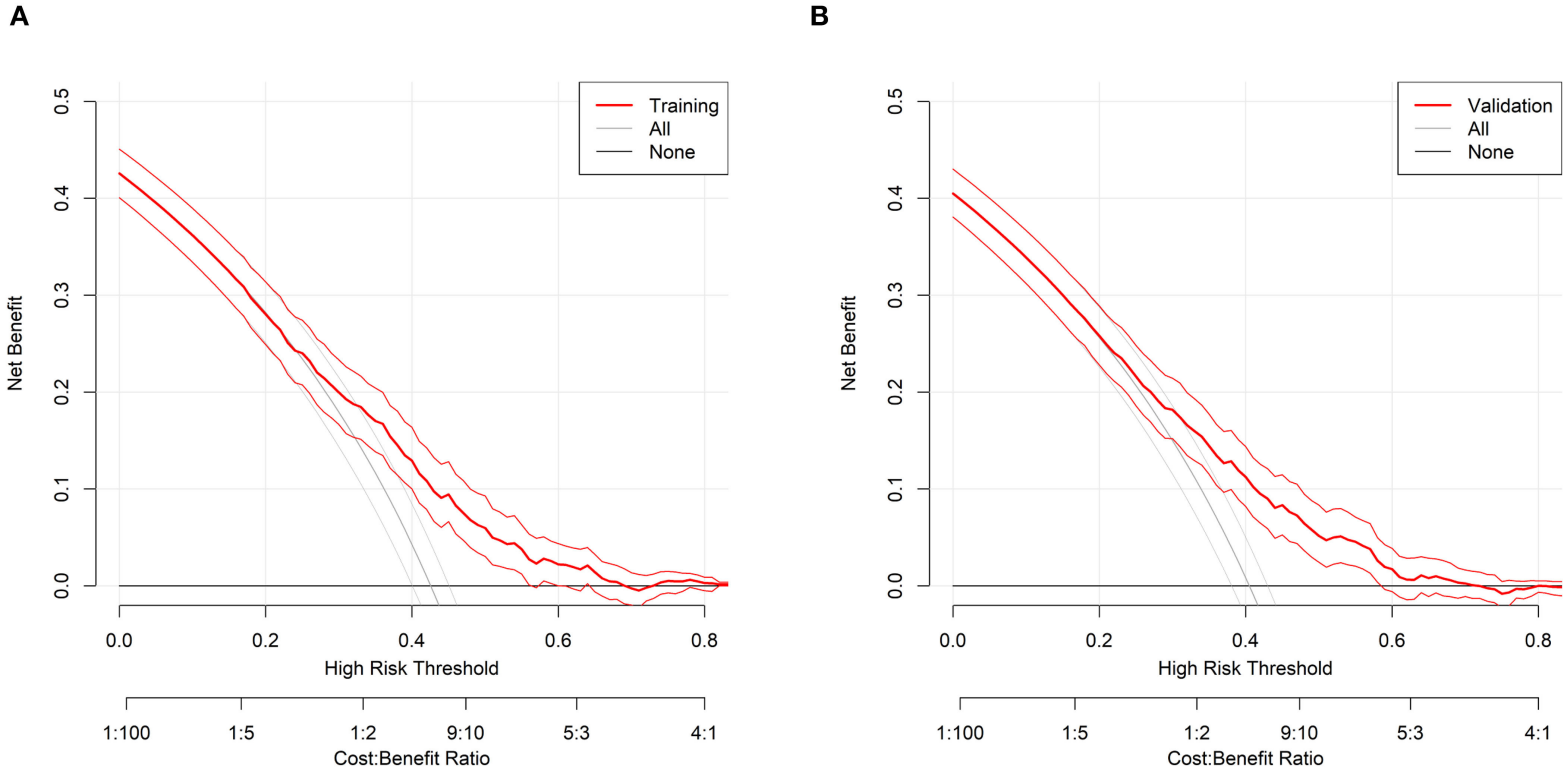
Decision analysis curve for the nomogram in the two groups. **(A)** The training group; **(B)** the validation group. Decision analysis curve is plotted by different risk threshold against net benefit. There are two lines for reference: A gray line indicates treated-for-all scheme, and a black line indicates treated-for-none scheme.

The Brier scores were both 0.22 in the training and validation groups, and the Brier-scaled scores were 8.66 and 9.43%, respectively (Table 4). The area under curve (AUC) values were 0.67 (95% CI: 0.64–0.70, Figure 4A) in the training group and 0.68 (95% CI: 0.66–0.71, Figure 4B) in the validation group. The corresponding discrimination slopes were 0.09 (Figure 5A) and 0.09 (Figure 5B), respectively. Figure 6 shows density curve, and it demonstrated that an acceptable separation was observed between participants with and without medical disputes in both groups. The calibration plots of the nomogram showed that the intercept was 0.00 (Figure 7A) in the training group and −0.06 (Figure 7B) in the validation group, and calibration slopes were 1.00 and 1.05, respectively. The Hosmer and Lemeshow test showed that the *P*-value was both 0.26 in the two groups. The study also presented model's specificity, sensitivity, accuracy, and threshold in both groups. The above results showed that the nomogram had acceptable discriminative ability and favorable calibrating ability. In addition, the decision curve analysis demonstrated the nomogram's positive clinical application value in the training (Figure 8A) and validation (Figure 8B) groups.

**Table 4:** Effectiveness of the model to predict probability of hospital administrators encountered medical disputes at work.

**Performance measure**	**Training group**	**Validation group**
**Overall**		
Brier score	0.22	0.22
Brier_scaled_ score	8.66%	9.43%
*R^2^*	0.11	0.13
**Discrimination**		
C-index (95% CI)	0.67 (0.64–0.70)	0.68 (0.66–0.71)
Discrimination slope	0.09	0.09
**Calibration**		
Intercept	0.00	−0.06
Calibration slope	1.00	1.05
H–L test	0.26	0.26
**Model usefulness**		
Sensitivity	79.41%	70.91%
Specificity	47.18%	57.05%
Accuracy	60.90%	62.67%
Best threshold	35.94%	38.83%

R^2^, R square; C-index, Area under the curve index; CI, confident interval; H–L test, Hosmer and Lemeshow test.

### Risk stratification

In terms of the optimal threshold (35.94% in the training group and 38.83% in the validation group), and to easily remember and conduct the risk stratification, this study defined that participants with a predicted probability of <30.00% were categorized into the low-risk group, participants with a predicted probability of 60.00% (double of the optimal threshold) or above were classified to the high-risk group, and a predicted probability of 30.00–60.00% belonged to the moderate-risk group (Table 5). The predicted probabilities of medical disputes were 23.82% in the low-risk group, 43.21% in the moderate-risk group, and 67.41% in the high-risk group, respectively, which were extremely close to the actual rates in the three risk groups. Notably, individuals in the low-risk group were 2.83 time less likely to have medical disputes as compared to those in the high-risk group (*P* < 0.001).

**Table 5:** Risk stratification of all participants based on the optimal threshold in the nomogram.

**Risk group**	**Participants (*n* = 2,716)**	**Predicted probability %**	**Actual probability**	***P***
Low risk (<30.00%)	585	23.82	21.54% (126/585)	<0.001
Moderate risk (30.00–60.00%)	1,770	43.21	43.16% (764/1,770)	
High risk (≥60.00%)	361	67.41	65.93% (238/361)	

## Discussion

### Main findings of the study

This study investigated the relationship between medical disputes and hospital legal constructions and further to build a nomogram to categorize participants at varying risk probabilities of experiencing medical disputes specifically among hospital administrators. According to the findings, there was a strong correlation between medical disputes and gender, professional ranks, duration of service, knowledge of hospital charters, and the construction status of hospital rule of law. These five features were included in the nomogram. The nomogram demonstrated positive predictive effectiveness based on the discriminative and calibrating ability, and a web calculator was developed to support model utility.

In addition, this study showed that when the number of laws or regulations training courses organized by hospitals was above six times a year, the incidence of medical disputes could start to decrease, and it could remarkably decline when the number of laws or regulations training courses was above 11 times a year (about once a month). Furthermore, only when participants had a perfectly clear understanding of knowledge on hospital rule of law, the incidence of medical disputes could considerably decline. These findings shed a light on the importance of laws or regulations training among hospital administers.

### Current construction status of hospital rule of law

Nearly, all participants' hospitals have established legal frameworks and performance appraisal systems related to the rule of law, but only about half of participants very clearly understood the duty of hospital law department, hospital charters, and the contents of legitimacy review in their hospitals, and also only half of participants reported that the construction status of hospital rule of law was very good. The aforementioned findings indicated that even though the majority of hospitals have established hospital law rules and legal regulations, the proportion of hospital administrators who understood these rules and regulations well was far from satisfactory, which in turn could help to explain the high incidence of medical disputes (41.53%) in the study.

The construction of the rule of law in the hospitals varies between countries (22). By enacting a law to combat corruption, the Canada health system's construction of the rule of law has been enhanced (23). Based on the root of corruption and inadequate governance structures in the health industry, the hospital rule of law in South Africa's healthcare system is unsatisfactory (24, 25). Insufficient rule of law construction has been linked to a number of factors, including ambiguous hospital charters, a lack of institutional accountability, conflicting rules, inadequate incentives for healthcare workers (26, 27), and a lack of sufficient individual capacity in policy planning and implementation (28, 29). As a result, this study made some recommendations to strengthen the construction of the rule of law in the hospitals. First, we should prioritize reviewing and amending the current laws regulations and developing a statutory minimum standard for medical practice in the hospitals (28). To achieve this, we suggested “Regulation on the Prevention and Handling of Medical Disputes” legal framework (30). In accordance with such legal framework, hospitals and medical staff should place a greater emphasis on providing medical care for patients, and it is the obligation of medical institutions to improve training on hospital legislation and professional ethics.

### Analysis of current studies on risk factors for medical disputes

Previous studies have demonstrated that misdiagnosis, a lack of informed consent, therapeutic plan errors, procedures that result in serious complications, and patient–physician mistrust were significantly associated with medical disputes (9, 31, 32). These factors may be very useful in guiding medical management. However, rather than hospital administrators, these studies were designed for clinical medical workers. Thus, it is possible that these factors may not be applicable to this population. In addition, constructive status of hospital rule of law was not assessed in the above studies.

In this study, we discovered that more medical disputes were significantly associated with male gender, higher professional rank, longer duration of service, less understanding of hospital charters, and poorer construction status of hospital rule of law. Studies also revealed that male doctors were more vulnerable to exposure to medical disputes as compared to females (2, 22, 33, 34), and the specialty grade of a doctor was proved to be relevant to medical disputes (22, 35). Several studies showed that hospital administrators' management skills would be constrained by their ignorance of the hospital's charter (28), making it impossible for them to swiftly and appropriately settle medical disputes (36). As a result, they might blame one another to avoid and shirk responsibility, when there were medical disputes between management divisions (36). Furthermore, it made sense that a shoddy construction of hospital rule of law might result in an increase in medical disputes. Because the medical practice of staff could not be well-restrained by the legal system in the hospitals, and the hospital's medical staff, at the same time, could not be adequately protected.

### Current available prediction model for hospital management

A chance-constrained model to predict stock management in hospital pharmacy (16) and a machine learning-based model to predict pharmaceutical ordering error identification (37) have both been published in previous studies. Two thousand twenty saw the development of a prediction model to measure patient satisfaction after incorporating sixteen different factors, including location, facility, sex, and age (19). A study that was conducted in 2021 also developed a prediction model to assess inpatient satisfaction in 16 large public hospitals (20). These predictive models are a clear and useful resource for clinical decision makers, hospital managers, and healthcare professionals. However, there were few models for predicting medical disputes. To the author's best knowledge, this model was the first to identify medical disputes specifically among medical administrators directly. The model was presented as the format of nomogram in the study that can be a useful tool for preventing medical disputes, guiding administrative management, and assisting decision-making.

### Preventive measures for medical disputes

According to previous studies, some steps could be taken to prevent medical disputes, such as updating dispute handling system, enhancing one's capacity to resolve disputes, and raising the standard of healthcare by strengthening one's professional abilities, service attitudes, and sense of responsibility among physicians (2, 9, 11, 38, 39). Our study also provided helpful additions to existing body of knowledge. The results of our study suggested that hospitals should work to improve rule of law training, particularly for men, so that they completely comprehended hospital charters, which would be very helpful to avoid and prevent medical disputes. More specifically, we recommended that training sessions for hospital rules and regulations should be at least six times annually and would better be once a month. That is because, as shown in this study, only when participants had a very clear understanding of knowledge on hospital rule of law, the incidence of medical disputes could remarkably decline. However, considering that it could be challenging for hospitals to conduct monthly legal training, we suggested the “hospital-department-personal” three-level legal training model. First, at the hospital level, it is recommended that hospitals should at least arrange one legal training for all hospital staff every 2 months; at the department level, it is also advised to organize training on laws and regulations at least six times a year. Thus, with the efforts of both the hospitals and departments, the total number of laws training could be up to 12 times a year. Second, hospitals should create effective strategies to attract administrators to engage in the mastery of hospital rules and regulations. Examples include making clear training management guidelines, awarding certificates for continuing education, and conducting online training about hospital rule and regulations. Notably, the network platform has no constraints on time, area, and resources; legal professionals are free to submit their courses there; and hospital staff members always have access to online legal training at their convenience. Third, since most laws and regulations are abstract and difficult to understand, it is advised, at the personal level, that administrators actively study and acquire knowledge of them, and it is important to examine laws and regulations on a regular basis.

To enable personalized application of preventive measures, risk stratification was achieved in the study, and individuals were divided into three risk categories. Participants in the high-risk group were 2.83 time more likely to have medical disputes as compared to those in the low-risk group. Therefore, individuals in the high-risk group should receive additional training in hospital rule of law, according to the nomogram. After training, their understanding of the contents of legal construction should be evaluated, and their participation in courses and exam performance should be connected to their salaries. While participants in the low-risk group may only need to attend training as normal, those in the moderate-risk group should continue to strengthen their self-study on the construction of the rule of law in their hospital.

Along with the above legal training courses and series of measures for various risk groups, hospital organizational management should also receive attention. First, it is necessary to clearly define each administrative department's obligation to avoid escape and shirk responsibility. Second, the role of hospital law department should be paid more emphasis in guiding hospital management, and its jobs should be supported by other departments. Third, the hospital law department needs greater funding so that they have the manpower and resources to plan and carry out a number of initiatives, such as inviting legal experts and organizing training courses on rule of law. Lastly, to encourage hospital staff to actively engage in the construction of hospital rule of law, it is also justified to establish clear and intelligible hospital charters and rule of law. Consequently, it is significant to strengthen the management and organizational capabilities of hospitals and further to increase staff compliance with regard to the construction of the rule of law within the hospitals. Notably, building the rule of law in the hospitals, especially tertiary hospitals, is an essential component of building the rule of law in China and a key component of building a healthy China.

## Limitations

The study has several drawbacks. First, medical disputes are complicated events that are the result of combination of a variety of factors, but this study only took seventeen variables into account and some other significant risk factors, such as the specialty of clinic, were not collected. Second, because this study is a cross-sectional analysis in nature, more research is necessary to determine the causal relationship between medical disputes and factors. Third, enrolled participants only included hospital administrators, so the applicability and generalization of the prediction model need further validations in other healthcare workers. In future, healthcare workers and patients should be included in the research to identify more comprehensive preventive strategies for medical disputes, which would contribute to the systematic construction of hospital rule of law.

## Conclusion

Medical dispute is prevailing among hospital administrators, and it can be reduced by the effective constructions of hospital rule of law. This study proposes a novel nomogram to predict the likelihood of medical disputes specifically among administrators in tertiary hospitals, and a web calculator can be available at https://ymgarden.shinyapps.io/Predictionofmedicaldisputes/. The nomogram may be a useful tool to evaluate the risk probability of medical disputes and further to guide administrative management and assist decision-making. To assist reduce medical disputes, more emphasis should be placed on helping participants in the high-risk group, such as enabling them to better understand hospital charters and encouraging the construction of the rule of law in their hospitals.

## Data availability statement

The raw data supporting the conclusions of this article will be made available by the authors, without undue reservation.

## Ethics statement

The studies involving human participants were reviewed and approved by Hunan Provincial Health Commission, an official health management organization in China, approved the study protocol. The patients/participants provided their written informed consent to participate in this study.

## Author contributions

MY and YaC were responsible for conception and design and involved in drafting the manuscript. MY and YZ provided statistical collection and analysis. KZ assisted in the statistical collection. YuC, XZ, JW, WC, and LW were involved in reviewing the manuscript. All authors revised the manuscript critically and approved the final version for submission.

## Funding

This study was supported by the National Social Science Foundation of China (Grant No. 21STA052) and the Project of the Health Commission of Hunan Province.

## Conflict of interest

The authors declare that the research was conducted in the absence of any commercial or financial relationships that could be construed as a potential conflict of interest.

## Publisher's note

All claims expressed in this article are solely those of the authors and do not necessarily represent those of their affiliated organizations, or those of the publisher, the editors and the reviewers. Any product that may be evaluated in this article, or claim that may be made by its manufacturer, is not guaranteed or endorsed by the publisher.
