# Supplementary material for: Association between hospital legal constructions and medical disputes: A multi-center analysis of 130 tertiary hospitals in Hunan Province, China
Source: Front Public Health. 2022 Sep 7;10:993946. doi: 10.3389/fpubh.2022.993946 (PMC9490230; doi:10.3389/fpubh.2022.993946)
Supplement: Supplementary file 1 [file Data_Sheet_1.PDF]

Construction Status of Hospital Rule of Law among Administrators Working in Tertiary Hospitals

Dear Participants,

Your participation in this survey on hospital legal construction is cordially welcomed. The survey seeks to investigate the construction status of hospital rule of law among administrators working in tertiary hospitals, as well as to discover pertinent risk factors associated with medical disputes. It should be noted that effective preventative measures for medical disputes can be carried out individually with the help of significant variables and prediction models.

Only tertiary hospital administrators were eligible to take the survey. You are requested to complete the survey on a completely voluntary and anonymous basis, and you are free to decline at any moment. It takes about 3 minutes to complete and the collected information is only used for research. Please answer each question truthfully, taking into account your current circumstances. Only after all of the survey's questions have been addressed can it be submitted.

Thank you for your assistance!

Sincerely,

Yanlin Cao
Institute of Medical Information and Library, Chinese Academy of Medical Sciences and Peking Union Medical College, Beijing, 100020, People's Republic of China.
E-mail: cao.yanlin@imicams.ac.cn.

Statement

☐ I have read and understood the informed consent and volunteer to participate in the survey.

☐ I decline to participate in this study.

Questions

1. What is your gender?

☐ Male

☐ Female

2. How old are you?

☐ <30

☐ 30-39

☐ 40-49

☐ ≥50

3. What is your professional rank?

☐ Senior

☐ Deputy senior

☐ Intermediate

☐ Junior

☐ None

4. What is your length of service in the hospital?

☐ <6

☐ 6-10

☐ 11-15

☐ 16-20

☐ >20

5. What is the hospital type of your hospital?

☐ Public hospital

☐ Private hospital

6. What is the hospital category of your hospital?

☐ General hospital

☐ Traditional Chinese Medicine (TCM) general hospital

☐ Specialized hospital

☐ Other hospital

7. What is the hospital level of your hospital?

☐ Class A tertiary hospital

☐ Class B tertiary hospital

☐ Other tertiary hospital

8. Are your hospital located in provincial capital?

☐ Yes

☐ No

9. Do you work in a department that provides window services to patients?

☐ Yes

☐ No

10. Have your hospital established law frameworks?

☐ Yes

☐ No

☐ Not clear

11. Do you understand the duty of hospital law department?

☐ Perfectly clear

☐ Clear

☐ Unclear

☐ None

12. How many times does the hospital organize training on laws or regulations?

☐ ≤1

☐ 2-3

☐ 4-6

☐ 7-10

☐ ≥11

13. How many times do you attend the hospital training of laws or regulations each year?

☐ 0

☐ 1-2

☐ 3-4

☐ ≥5

14. Do you understand the hospital charter of your hospital?

☐ Perfectly clear

☐ Clear

☐ Unclear

☐ No hospital charters

☐ Not at all

15. Have your hospital established a performance appraisal system related to the rule of law?

☐ Yes

☐ No

16. Do you understand the contents of legitimacy review in your hospital?

☐ Perfectly clear

☐ Clear

☐ Unclear

☐ Not at all

17. Have you ever encountered medical disputes in your work?

☐ No

☐ Yes

18. What is the construction status of the rule of law in your hospital?

☐ Very good

☐ Good

☐ Neither good nor bad

☐ Bad

☐ Very bad

19. What is the name of your hospital? (Filling in the blanks)

____

Notes: All of these questions are self-reported. Hospital charters are law frameworks for hospitals made up of state legislation, administrative norms, and hospital rules and regulations. Performance appraisal system mainly refers to performance of job responsibilities, completion of annual work, and work attendance, which can directly affect earnings, awards, and academic or job title assessments. The contents of legitimacy review refer to major policy measures for hospital, all kinds of master plans, major hospital investment projects and the disposal of state-owned assets, or other major matters that require decision-making by the hospital. Medical disputes

include doctor-patient disputes, disputes between patients or their families and hospital staff, and other disputes related to medical works.
